# BMAL1 Disrupted Intrinsic Diurnal Oscillation in Rat Cerebrovascular Contractility of Simulated Microgravity Rats by Altering Circadian Regulation of miR-103/Ca_V_1.2 Signal Pathway

**DOI:** 10.3390/ijms20163947

**Published:** 2019-08-14

**Authors:** Li Chen, Bin Zhang, Lu Yang, Yun-Gang Bai, Ji-Bo Song, Yi-Ling Ge, Hong-Zhe Ma, Jiu-Hua Cheng, Jin Ma, Man-Jiang Xie

**Affiliations:** 1 Department of Aerospace Physiology, Key Laboratory of Aerospace Medicine of Ministry of Education, Fourth Military Medical University, Xi’an 710032, China; 2 Department of Physiology, Fourth Military Medical University, Xi’an 710032, China; 3 First Cadet Brigade, Fourth Military Medical University, Xi’an 710032, China

**Keywords:** simulated microgravity, vascular contractility, L-type Ca_V_1.2 channel, circadian regulation, miRNA

## Abstract

The functional and structural adaptations in cerebral arteries could be one of the fundamental causes in the occurrence of orthostatic intolerance after space flight. In addition, emerging studies have found that many cardiovascular functions exhibit circadian rhythm. Several lines of evidence suggest that space flight might increase an astronaut’s cardiovascular risks by disrupting circadian rhythm. However, it remains unknown whether microgravity disrupts the diurnal variation in vascular contractility and whether microgravity impacts on circadian clock system. Sprague-Dawley rats were subjected to 28-day hindlimb-unweighting to simulate the effects of microgravity on vasculature. Cerebrovascular contractility was estimated by investigating vasoconstrictor responsiveness and myogenic tone. The circadian regulation of Ca_V_1.2 channel was determined by recording whole-cell currents, evaluating protein and mRNA expressions. Then the candidate miRNA in relation with Ca^2+^ signal was screened. Lastly, the underlying pathway involved in circadian regulation of cerebrovascular contractility was determined. The major findings of this study are: (1) The clock gene BMAL1 could induce the expression of miR-103, and in turn modulate the circadian regulation of Ca_V_1.2 channel in rat cerebral arteries at post-transcriptional level; and (2) simulated microgravity disrupted intrinsic diurnal oscillation in rat cerebrovascular contractility by altering circadian regulation of BMAL1/miR-103/Ca_V_1.2 signal pathway.

## 1. Introduction

Postflight orthostatic intolerance has been considered as one of the major adverse effects after spaceflight, in which multiple mechanisms have been reported to be implicated, such as hypovolemia, altered neurohumoral regulation and aerobic capacity, alterations in baroreflex sensitivity, and cardiovascular dysfunction [1,2]. Human studies from real microgravity (spaceflight) or simulated microgravity (head-down tilt bed rest) have revealed that impaired autoregulation of cerebral vasculature contributes to the occurrence of postflight orthostatic intolerance. In addition, ground-based animal studies with tail-suspended (SUS) hindlimb-unweighting rat models have clearly demonstrated that simulated microgravity induces the hypertrophic remodeling in cerebral arteries including increased media thickness, augmented myogenic tone, enhanced arterial reactivity, and impaired endothelial function [3]. All these findings suggest that functional and structural adaptations in cerebral arteries are fundamental causes in the occurrence of postflight orthostatic intolerance, but the underlying mechanisms remain to be fully clarified [1,3].

Emerging studies have found that many cardiovascular functions exhibit biological circadian rhythm to adapt to Earth’s cycling environment. For example, the incidence of ventricular arrhythmias, cardiac sudden death, and myocardial infarction exists apparent circadian changes with the peaking time in the early morning. In addition, blood pressure, heart rate, vascular contractility, endothelial function, sympathetic nerve activity, and platelet aggregability also display obvious diurnal variation during the course of about a 24-h cycle [4]. Cardiovascular intrinsic timekeeping is driven by a central clock and peripheral tissue clocks. The central clock is located at the suprachiasmatic nuclei (SCN) of the hypothalamus, which functions as the master pacemaker by synchronizing the peripheral clocks in accordance with environmental fluctuations [5]. At the molecular level, mammalian clocks are composed of autoregulated transcriptional-translational feed-back and feed-forward loops, with a period close to 24-h to drive the gene oscillation. BMAL1 (Brain and muscle aryl-hydrocarbon receptor nuclear translocator-like 1) and CLOCK (Circadian Locomotor Output Cycles Kaput) are two main elements that bind to the promoters of the Period (Per1 and Per2) and Cryptochrome (Cry1 and Cry2) genes [5]. Upon accumulation in the cytoplasm, the protein products of Per and Cry translocate to the nucleus and inhibit CLOCK/BMAL1-mediated transcription as negative elements, which leads to repression of their own transcription. This core loop is also interconnected with additional positive and negative regulatory loops, including nuclear receptors such as REV-ERBα (NR1D1, nuclear receptor subfamily 1, group D, member 1), RORα (RAR-related orphan receptor alpha), and PPARs (Peroxisome proliferator-activated receptors). These clock genes control numerous target genes (clock-controlled genes, CCGs) and work as transcription factors to produce the diurnal rhythmic expression in approximately 10% of genomic genes, which in turn provide the diurnal variation for cardiovascular function [6].

Diurnal variation for cardiovascular function is considered to be mainly attributable to the day/night time variances in vascular contractility [7,8]. Vascular smooth muscle cells (VSMCs) are major components in the vessel wall, and their contraction is an important physiological process for maintaining cardiovascular homeostasis in response to environmental cues. The fundamental pathway for smooth muscle contraction is the increase of intracellular Ca^2+^ concentration [5,9]. Increased Ca^2+^ binds to CaM (calmodulin) and this complex activates MLCK (myosin light chain kinase), which elicits vascular contraction by phosphorylation of MLC (myosin light chains). A secondary pathway for vascular contraction is the RhoA/Rho kinase pathway by modulating Ca^2+^ sensitization [7,10]. In response to contractile stimuli, the small GTPase RhoA activates its downstream effector Rho kinase, which in turn initiates contraction by inhibition of MLCP (myosin light chain phosphatase). Interestingly, it has been demonstrated that the expression and activity of ROCK2 (Rho-associated kinase 2) exhibits a diurnal oscillatory with that of MLC phosphorylation and myofilament Ca^2+^ sensitization, which are provoked by the clock gene BMAL1 and RORα in VSMCs [7,10]. The L-type (large or long-lasting) voltage-dependent Ca^2+^ channel (VDCC, Ca_V_1.2) is the main Ca^2+^ influx pathway in VSMCs and then is considered as the primary determinant of VSMC contractility and vascular tone [5,9,11]. However, it is unknown whether there is an existence of intrinsic circadian regulation of Ca_V_1.2 channel for vascular contractility. Furthermore, the molecular mechanisms underlying the Ca_V_1.2-related pathway responsible for vascular diurnal contraction are completely unclear.

Among the zeitgebers (external time-giving cues), light is the major input into the central clock, which drives and coordinates the internal oscillators in various peripheral tissues through behavioral and neurohumoral transmitters [12,13]. In addition, exercise, jetlag, shift work, temperature, feeding, and nutritional signals can also be sensed by clock system and then influence the circadian rhythm. Recently, several lines of evidence from real spaceflight or simulated microgravity studies suggest that gravitational change might be another important cause that influences circadian oscillation [14,15]. For example, the rhythmicity of an astronaut’s body temperature significantly decreased in Russian Mir station flight as compared with that on Earth [16]. Long-term exposure to microgravity disturbs the astronaut’s autonomic nervous functions and intrinsic cardiovascular functions evaluated by heart rate variability (HRV) [17]. In addition, human head-down tilt bed rest experiments indicate that simulated microgravity disrupts the diurnal variation in heart rate, blood pressure [18]. Circadian disruption could increase an astronaut’s cardiovascular risks and cause a decreased ability to effectively and efficiently perform tasks. However, it is not clear whether microgravity could disrupt the diurnal variation in vascular contractility. Furthermore, how the circadian clock is influenced by microgravity, and then generates the altered cardiovascular circadian variation, remains poorly understood.

The purpose of the present work was (1) to confirm whether simulated microgravity influences the diurnal contractility in rat cerebral arteries; (2) to investigate the circadian regulation of Ca_V_1.2 channel (the primary determinant of VSMC contractility) in cerebral VSMCs by recording whole-cell currents, evaluating protein and mRNA expressions; and (3) to screen the candidate miRNA (the upstream signaling) in relation with Ca^2+^ signaling in VSMCs and then identify the involvement of the BMAL1/miRNA/Ca_V_1.2 pathway.

## 2. Results

### 2.1. General Data

There were no significant differences in the initial or final body weights between control (CON) and tail-suspended (SUS) rats, indicating a normal growth rate during simulated microgravity. However, either the wet weights of the left soleus or the ratio of soleus/body weight significantly decreased in SUS as compared with that in CON rats, which suggested the deconditioning effects of simulated microgravity (Table 1).

### 2.2. The Diurnal Variation of Vasoconstrictor Responsiveness to 5-HT Was Suppressed in Middle Cerebral Arteries of Simulated Microgravity Rats

**Figure 1:**
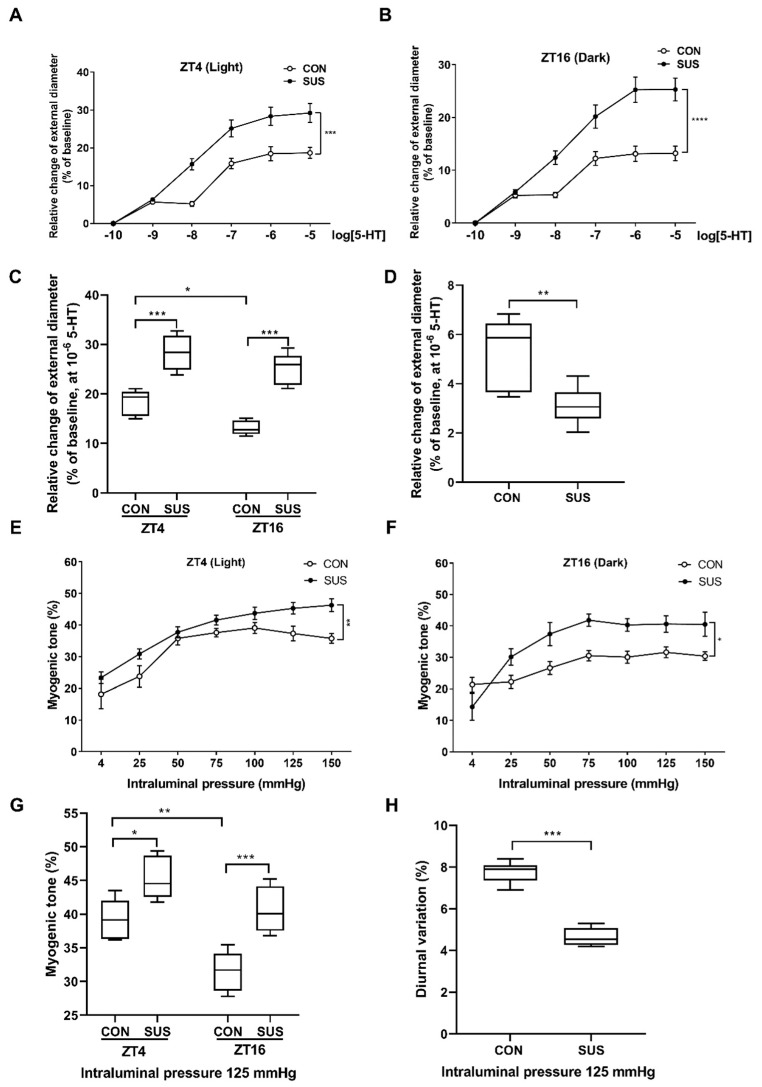
Diurnal rhythm of vasoconstrictor responsiveness to 5-HT and myogenic tone in middle cerebral arteries isolated from CON and SUS rats. The concentration-response curves were determined by the cumulative superfusion of 5-hydroxytryptamine (5-HT, 10^−10^ to 10^−5^ M) in middle cerebral arteries isolated from CON and SUS rats at ZT4 (**A**) and ZT16 (**B**), respectively. The contractile responses in response to 10^−6^ M 5-HT were markedly increased at both ZT4 and ZT16 in SUS rats as compared with that in CON rats (**C**). The diurnal variation in vasoconstrictor responsiveness (the difference value between ZT4 and ZT16 level) significantly decreased in SUS rats as compared with that in CON rats (**D**). The myogenic tone of middle cerebral arteries isolated from CON and SUS rats was calculated when intraluminal pressure ranging from 4 to 150 mmHg at ZT4 (**E**) and ZT16 (**F**), respectively. The myogenic tone markedly increased at both ZT4 and ZT16 in SUS rats as compared with that in CON rats when intraluminal pressure was 125 mmHg (**G**). The diurnal variation in myogenic tone (the difference value between ZT4 and ZT16 level) significantly decreased in SUS rats as compared with that in CON rats (**H**). Data are presented as box plots and 5th and 95th percentiles. *n* = 6, *t*-test, * *p* < 0.05, ** *p* < 0.01, *** *p* < 0.001. CON: 28-day simultaneous control rats; SUS: 28-day tail-suspended rats.

Consistent with a previous report in superior mesenteric arteries [10], the contractile responses to 5-HT stimulation in rat middle cerebral arteries were higher at zeitgeber time 4 (ZT4) (the subjective light period) than at ZT16 (the subjective dark period) in CON rats (Figure 1A–C), suggesting that the cerebrovascular contraction displayed a diurnal rhythm with the peak at light phase. In addition, the contractile responses were markedly increased at both ZT4 and ZT16 in SUS as compared with that in CON rats (Figure 1A–C), which is similar to our previous report [19]. However, simulated microgravity significantly suppressed the diurnal variation (the difference value between ZT4 and ZT16 level) in responses to 5-HT as compared with that in CON rats (Figure 1D).

### 2.3. The Diurnal Variation of Myogenic Tone Was Attenuated in Middle Cerebral Arteries of Simulated Microgravity Rats

Step increases in intraluminal pressure induced an increase in luminal diameter under passive and active conditions, respectively (not shown). Consistent with a previous report [10], the myogenic tone in rat middle cerebral arteries was higher at ZT4 than at ZT16 in CON rats (Figure 1E–G). In addition, the myogenic tone significantly increased at both ZT4 and ZT16 in SUS as compared with that in CON rats, which is similar to our previous report [19]. However, simulated microgravity significantly attenuated the diurnal variation of myogenic tone as compared with that in CON rats (Figure 1H).

### 2.4. Circadian Activities and Protein Expression of Ca_V_1.2 Channel, but Not mRNA Level, Were Altered in Cerebral Arteries of Simulated Microgravity Rats

**Figure 2:**
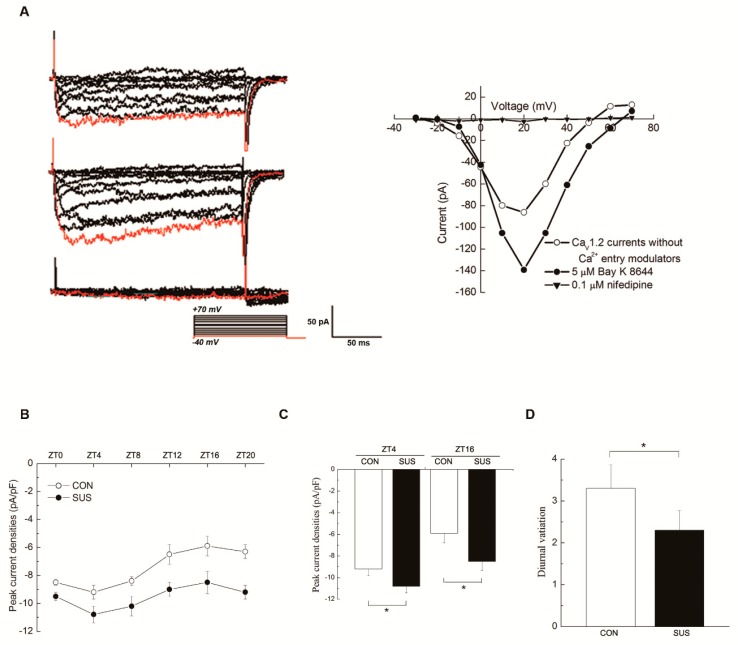
Circadian rhythm of Ca_V_1.2 activities in cerebrovascular SMCs isolated from CON and SUS rats at six different time points. (**A**) The representative families of inward currents recorded in cerebrovascular SMCs without Ca^2+^ entry modulators, in the presence of agonist Bay K 8644 or antagonist nifedipine in the bath solution. (**B**) The peak current densities of Ca_V_1.2 channel at +20 mV showed a circadian oscillation with the peak activities at ZT4 (the subjective light) and the trough activities at ZT16 (the subjective dark) in CON rats. (**C**) The peak current densities of Ca_V_1.2 markedly increased at both ZT4 and ZT16 in SUS rats as compared with that in CON rats. (**D**) The diurnal variation in Ca_V_1.2 activities (the difference value between ZT4 and ZT16 level) significantly decreased in SUS rats as compared with that in CON rats. Data are presented as box plots and 5th and 95th percentiles. *n* = 10, *t*-test or two-way ANOVA and Tukey’s multiple comparisons test, * *p* < 0.05, CON: 28-day simultaneous control rats; SUS: 28-day tail-suspended rats.

The activities and expressions of Ca_V_1.2 channel in cerebral arteries were investigated at six different time points (ZT0, 4, 8, 12, 16, and 20). As shown in Figure 2A, 5 μM Bay K 8644 (the specific agonist) significantly increased the inward currents, whereas 0.1 μM nifedipine (the specific antagonist) markedly suppressed the inward currents, which obviously indicated the property of Ca_V_1.2 channel. The peak current densities at +20 mV showed a circadian oscillation with the peak at ZT4 and the trough at ZT16 in CON rats (Figure 2B), which corresponds to the diurnal variation of cerebrovascular contractility in CON as shown above (Figure 1). In addition, the activities of Ca_V_1.2 channel were markedly increased at both ZT4 and ZT16 in SUS as compared with that in CON rats (Figure 2C), which is similar to our previous report [9]. However, simulated microgravity significantly decreased the diurnal variations of Ca_V_1.2 activities as compared with that in CON rats (Figure 2D).

**Figure 3:**
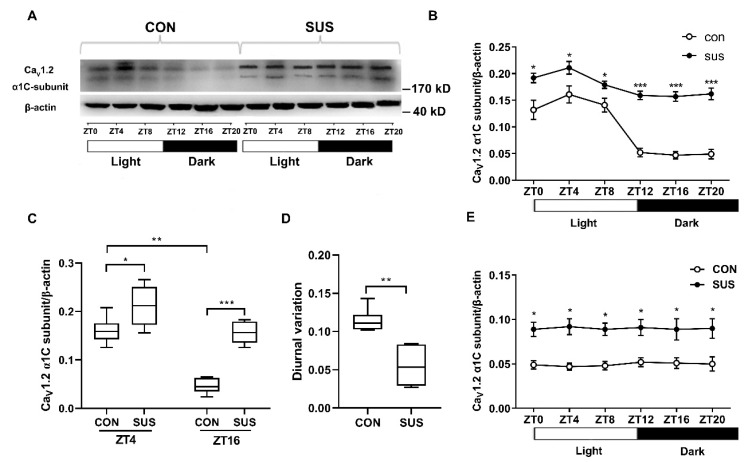
Circadian expression of Ca_V_1.2 α1C-subunit in cerebral arteries isolated from CON and SUS rats at six different time points. (**A**) The representative protein expressions of Ca_V_1.2 α1C-subunit are shown. (**B**) The mean data of protein expression display a circadian oscillation with the acrophase at subjective light-time and the trough at subjective dark-time. (**C**) The protein expression of Ca_V_1.2 α1C-subunit markedly increased at both ZT4 and ZT16 in SUS rats as compared with that in CON rats. (**D**) The diurnal variation in protein expression of Ca_V_1.2 α1C-subunit (the difference value between ZT4 and ZT16 level) significantly decreased in SUS rats as compared with that in CON rats. (**E**) both Ca_V_1.2 mRNA expressions of cerebral arteries in SUS and CON rats remained constant throughout the course of a day. Data are presented as box plots and 5th and 95th percentiles. *n* = 6, *t*-test or two-way ANOVA and Tukey’s multiple comparisons test, * *p* < 0.05, ** *p* < 0.01, *** *p* < 0.001 as compared with CON. CON: 28-day simultaneous control rats; SUS: 28-day tail-suspended rats (the full-length Western blots for Figure 3A are shown in the Supplementary Information).

Similar to Ca_V_1.2 activities, protein expression of Ca_V_1.2 α1C-subunit in cerebral arteries exhibited a daily rhythm with the acrophase at light-time and the trough at dark-time (Figure 3A,B). In addition, the protein expressions were significantly increased at both ZT4 and ZT16 in SUS as compared with that in CON rats (Figure 3B,C). However, simulated microgravity significantly decreased the diurnal variations in protein expression of Ca_V_1.2 α1C-subunit as compared with that in CON rats (Figure 3D). Interestingly, both Ca_V_1.2 α1C-subunit mRNA expressions of cerebral arteries in SUS and CON rats remained constant throughout the course of a day (Figure 3E), which was different from circadian oscillations of Ca_V_1.2 activities and protein expressions. The difference of circadian activities between protein expression and mRNA expression implies that there might be a functional post-transcriptional regulation.

### 2.5. MicroRNA-103 Is the Upstream Signaling in the Circadian Output Regulation of Ca_V_1.2 Channel

**Figure 4:**
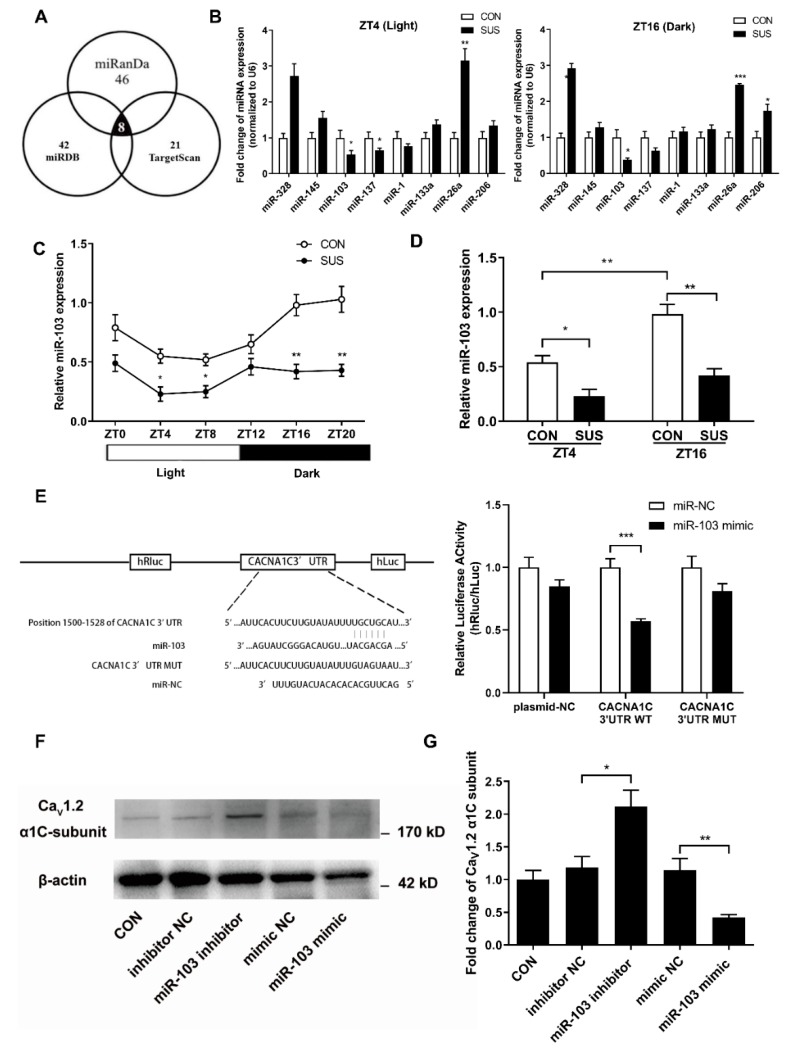
Identification of Ca_V_1.2 α1C-subunit as the down target of miR-103 in VSMCs. (**A**) Computational prediction of Ca^2+^ signaling-related miRNAs by evaluating the published literatures and the prediction software programs of miRanDa, miRDB, and Targetscan. (**B**) qRT-PCR was performed to examine the mRNA levels of eight candidate miRNAs in cerebral VSMCs of SUS rats (*n* = 3/group). (**C**) More samples were used to further confirm that miR-103 expression of cerebral arteries showed a circadian rhythm with a higher level during the subjective night than the subjective light in CON and SUS rats (*n* = 6). (**D**) The expression of miR-103 markedly decreased at both ZT4 and ZT16 in SUS rats as compared with that in CON rats. (**E**) Schematic diagram of the presumptive binding sequences of miR-103 to CACNA1C 3′-UTR based on the TargetScan database prediction. Dual-luciferase activity assay was performed in A7r5 cells by co-transfecting miR-103 mimic with psiCHECK^TM^-2 vectors containing WT/MUT CACNA1C 3′-UTR. Renilla luciferase activity was normalized by Firefly luciferase activity (hRluc/hLuc). (**F**,**G**) Western blotting was used to show that overexpression or inhibition of miR-103 by mimic or inhibitor transfection significantly decreased or increased the protein expression of Ca_V_1.2 channel in A7r5 cells, respectively. Data are presented as box plots and 5th and 95th percentiles. Each experiment in vitro was repeated three times, *t*-test or one(two)-way ANOVA and Tukey’s multiple comparisons test, * *p* < 0.05, ** *p* < 0.01, *** *p* < 0.001 as compared with CON (the full-length Western blots for Figure 4F are shown in the Supplementary Information).

To investigate the post-transcriptional regulation, eight miRNAs (miR-328, miR-145, miR-103. miR-137, miR-1, miR-133a, miR-26a, and miR-206) were narrowed to our choices for the candidate miRNA (Figure 4A). Only miR-103 expression in cerebral arteries of SUS rats significantly decreased at both ZT4 and ZT16, which corresponds to the markedly increased Ca_V_1.2 protein expression at both ZT4 and ZT16 in SUS as compared with that in CON rats (Figure 4B). Therefore, miR-103 was chosen as a possible negative regulator for Ca_V_1.2 channel. Cerebrovascular miR-103 expressions displayed a diurnal rhythm with higher levels during the subjective night, which was nearly anti-phase to the circadian rhythms of Ca_V_1.2 channel activities and protein expression as observed (Figure 4C,D). Furthermore, Dual Luciferase Reporter Assay System was applied to validate whether Ca_V_1.2 channel was the direct down target of miR-103 (Figure 4E). The plasmid (WT/MUT/NC) and miR-103 mimic were co-transfected into A7r5 VSMCs. Results show that miRNA-103 mimic only significantly suppressed the relative (hRluc/hLuc) luciferase activity of wild-type (WT) reporter of *CACNA1C* 3′-UTR, but not mutant (MUT) reporter or bland plasmid (plasmid-NC), which provided clear evidence that vascular miR-103 specifically targeted the 3′-UTR of Ca_V_1.2 α1C-subunit. Lastly, gain- and loss-function studies indicated that miR-103 inhibitor significantly increased, whereas miR-103 mimic decreased the protein expression of Ca_V_1.2 α1C-subunit in cultured VSMCs (Figure 4F,G). These results indicate that miR-103 negatively regulated the protein expression of Ca_V_1.2 α1C-subunit by direct binding to *CACNA1C* 3′-UTR at post-transcriptional level.

### 2.6. Circadian Expressions of BMAL1 Was Dampened in SCN and Cerebral Arteries of Simulated Microgravity Rats

**Figure 5:**
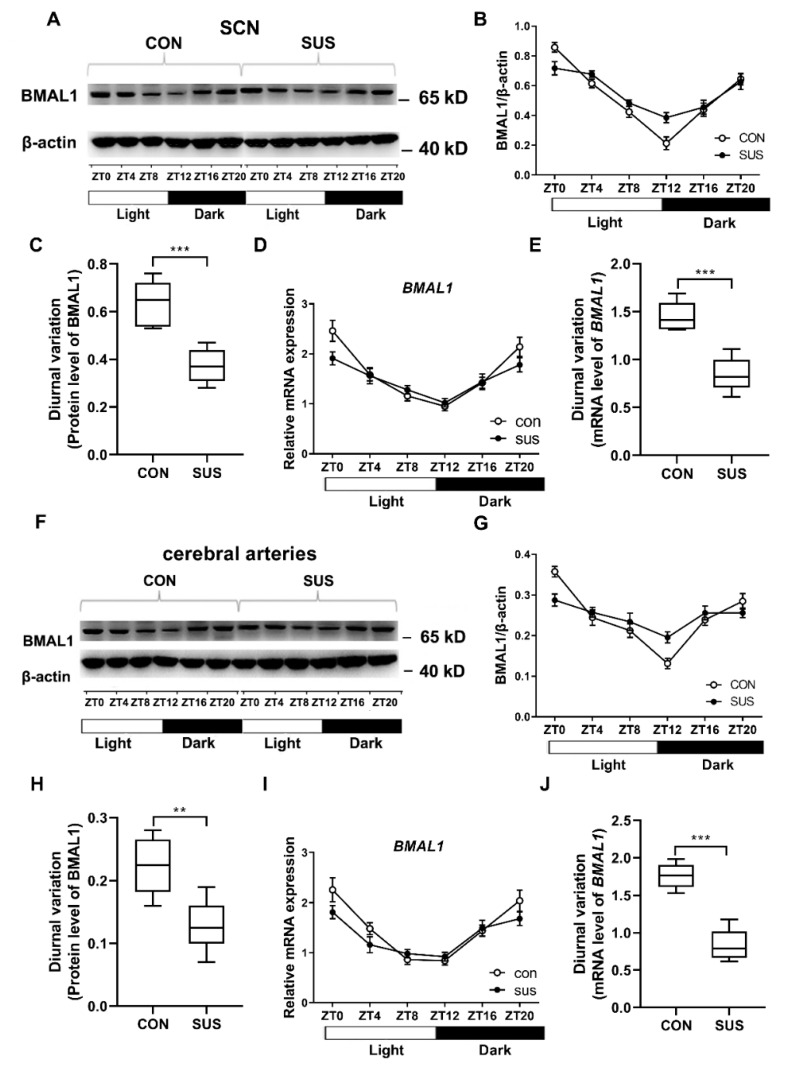
Circadian expression of clock gene BMAL1 in suprachiasmatic nuclei (SCN) and cerebral arteries isolated from CON and SUS rats at six different time points. (**A**) The representative protein expressions of BMAL1 in SCN are shown. The mean data of BMAL1 protein (**B**) and mRNA (**D**) expression in SCN display a circadian oscillation. The diurnal variation in BMAL1 protein (**C**) and mRNA (**E**) expression (the difference value between ZT0 and ZT12 level) in SCN significantly decreased in SUS rats as compared with that in CON rats. (**F**) The representative protein expressions of cerebrovascular BMAL1 are shown. The mean data of cerebrovascular BMAL1 protein (**G**) and mRNA (**I**) expression display a circadian oscillation. The diurnal variation in cerebrovascular BMAL1 protein (**H**) and mRNA (**J**) expression (the difference value between ZT0 and ZT12 level) significantly decreased in SUS rats as compared with that in CON rats. Data are presented as box plots and 5th and 95th percentiles. *n* = 6, *t*-test or two-way ANOVA and Tukey’s multiple comparisons test, ** *p* < 0.01, *** *p* < 0.001 as compared with CON. CON: 28-day simultaneous control rats; SUS: 28-day tail-suspended rats (the full-length Western blots for Figure 5A,F are shown in the Supplementary Information).

To further delineate the underlying mechanism, we next sought to investigate the core clock gene BMAL1 in rat SCN and cerebral arteries. Either in the central clock of SCN (Figure 5A–E) or in the peripheral clock of cerebral arteries (Figure 5F–J), BMAL1 protein (Figure 5A–C,F–H) and mRNA expression (Figure 5D,E,I,J) displayed a circadian oscillation with the peak at ZT0 and the trough at ZT12, which was the similar phase observed in vascular miR-103 expression (Figure 4C). However, simulated microgravity significantly attenuated the diurnal variations of BMAL1 protein (Figure 5C,E) and mRNA expression (Figure 5H,J) (the difference value between ZT0 and ZT12 level) in rat SCN and cerebral arteries.

### 2.7. BMAL1 Induced the Circadian Regulation of miR-103/Ca_V_1.2 Signal Pathway

**Figure 6:**
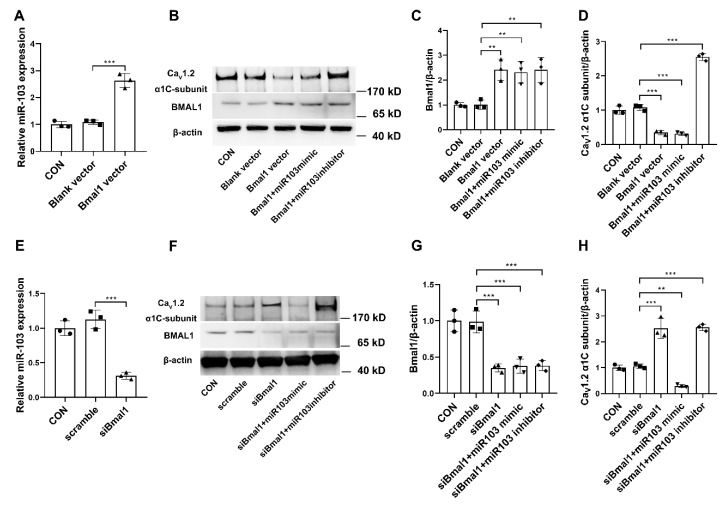
BMAL1 modulates the protein expression of Ca_V_1.2 α1C-subunit by miRNA-103 signal pathway. (**A**) The mRNA levels of miR-103 in A7r5 cells transfected with BMAL1 overexpression plasmid. The representative protein expressions of Ca_V_1.2 α1C-subunit and BMAL1 (**B**) and the mean data (**C**,**D**) with the treatment of miR-103 mimic and inhibitor in A7r5 cells transfected with BMAL1 overexpression plasmid, respectively. (**E**) the mRNA levels of miR-103 in A7r5 cells transfected with BMAL1 siRNA. The representative protein expressions of Ca_V_1.2 α1C-subunit and BMAL1 (**F**) and the mean data (**G**,**H**) with the treatment of miR-103 mimic and inhibitor in A7r5 cells transfected with BMAL1 siRNA, respectively. Data are presented as box plots and 5th and 95th percentiles. Each experiment was repeated three times, one-way ANOVA and Tukey’s multiple comparisons test, ** *p* < 0.01, *** *p* < 0.001 as compared with the control (the full-length Western blots for Figure 6B,F are shown in the Supplementary Information).

**Figure 7:**
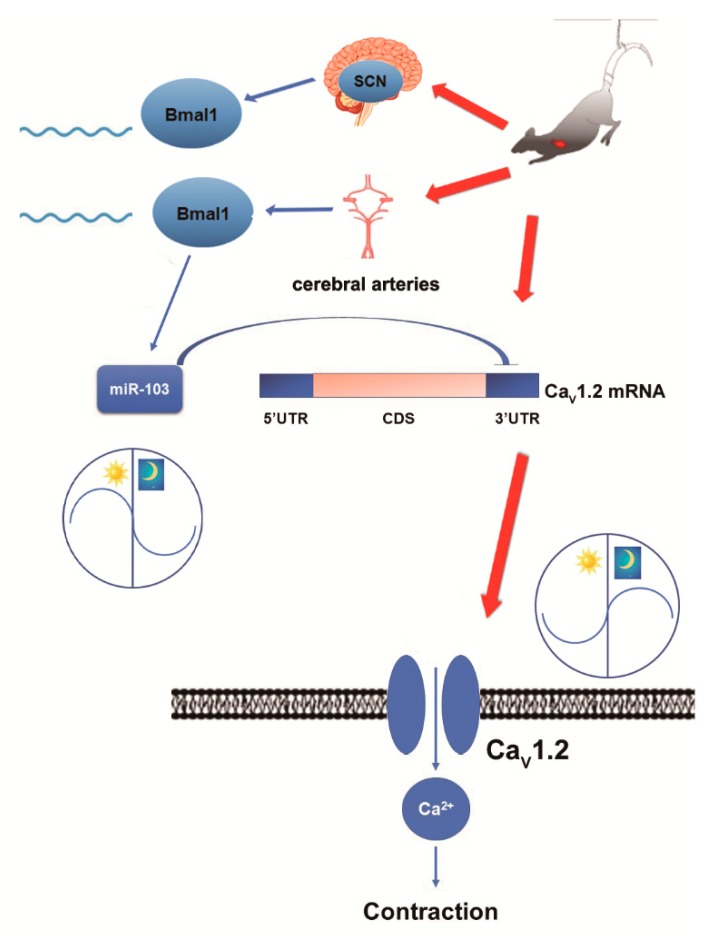
A schematic model of the circadian regulation of BMAL1/miR-103/Ca_V_1.2 signal pathway and the disruption of simulated microgravity.

Overexpression and silencing of BMAL1 were used to investigate the functional circadian regulation of miR-103/Ca_V_1.2 in cultured VSMCs. Overexpression of BMAL1 by plasmid vector significantly increased the mRNA levels of miR-103 (Figure 6A), while reducing the protein expressions of Ca_V_1.2 α1C-subunit (Figure 6B–D). Furthermore, the negative effects of BMAL1 on Ca_V_1.2 α1C-subunit could be abolished by the inhibition of miR-103 (Figure 6B–D). In contrast, silencing of BMAL1 by siRNA markedly decreased the mRNA levels of miR-103 (Figure 6E), whereas it increased the protein expressions of Ca_V_1.2 α1C-subunit (Figure 6F–H). Furthermore, miR-103 mimic could decrease BMAL1 silencing-induced protein expression of Ca_V_1.2 channel (Figure 6E,F). Collectively, our findings suggest that the expressions of the vascular Ca_V_1.2 were under the control of BMAL, probably through the miR-103 regulation (Figure 7).

The clock gene BMAL1, either in the central clock of SCN or the peripheral clock of cerebral arteries, can induce the expression of miR-103 and, in turn, modulate the circadian regulation of Ca_V_1.2 channel in rat cerebral arteries at post-transcriptional level. Simulated microgravity can alter the circadian regulation of BMAL1/miR-103/Ca_V_1.2 signal pathway, therefore disrupting intrinsic diurnal oscillation in rat cerebrovascular contractility.

## 3. Discussion

The major and novel findings of this study are: (1) Simulated microgravity disrupted the diurnal variation in rat cerebral arterial contractility; (2) the activities and protein expressions of Ca_V_1.2 channel, but not mRNA expression, exhibited a circadian regulation responsible for cerebrovascular contractility; and (3) Ca_V_1.2 channel was the direct down target of miR-103 in VSMCs, and the signal pathway of BMAL1/miR-103/Ca_V_1.2 was a novel mechanism underlying the circadian dysfunction in cerebrovascular contractility of simulated microgravity rats.

During spaceflight, astronauts are exposed to a microgravity environment which is dramatically different from that on Earth. Previous studies indicate that microgravity induces bone-loss, immune-suppression, cardiovascular dysfunction, and impaired secretion of hormone and neurotransmitter, which suggests that gravity exerts a significant impact on human body [1,2]. When exposed to microgravity, the hydrostatic gradients are lost throughout the vasculature, which induces a cephalad shift in fluid distribution from the lower part of the body towards the upper body [20]. Therefore, the absence of gravitational stimuli during spaceflight induces a number of adaptive changes in the cardiovascular system that may result in the occurrence of postflight orthostatic intolerance [1,3]. Microgravity-induced blood volume redistribution has been considered to be the initial trigger to cardiovascular dysfunction, however, which is a complex process involving diverse and complicated mechanisms [1,3].

Recently, evidence has indicated that there might be an integrated signaling network which could sense the microgravity signal and, in turn, modulate the circadian intrinsic timekeeping, including general behavior, hormone synthesis, body temperature, and metabolism [14,15]. For example, simulated microgravity enhanced the amplitude oscillations of BMAL1 clock gene in human keratinocytes with an apparently lower variability of REV-ERBα transcription. In contrast, recovery from simulated microgravity also increased the amplitudes and lengths of BMAL1 and Rev-erbα cycle periods [15]. By investigating vasoconstrictor responsiveness and myogenic tone, we found that there was an existence of diurnal variation in rat cerebrovascular contractility with the higher amplitude in the subjective light period(ZT4) and the lower in the subjective dark period(ZT16), which is similar to the previous reports of a circadian rhythm in vascular contractility with a peak at the beginning light phase in nocturnal animals [7,8,10]. Furthermore, we found that simulated microgravity markedly increased the cerebrovascular contractility at both ZT4 and ZT16, whereas significantly suppressed the intrinsic diurnal variation of rat cerebral vascular contractility. Therefore, microgravity-induced diurnal variation in cerebrovascular contractility may participate in the functional and structural adaptations in cerebral arteries, which causes the occurrence of postflight orthostatic intolerance.

The L-type Ca_V_1.2 channels mediate a voltage-dependent and depolarization-induced calcium influx, which are composed of a pore-forming α1C-subunit and auxiliary β, α2δ, and γ subunits [5,21]. Ca_V_1.2 channels in the retina were reported to be under circadian control with the higher current densities and α1C-subunit expressions at protein and mRNA level during the subjective dark period [13,22,23], which play an essential role in the neurotransmitter release from photoreceptors and other retinal neurons. Expression of Ca_V_1.2 α1C-subunit mRNA in SCN was rhythmic with the peaking during the late night, which was regulated by the clock gene REV-ERBα [24]. In embryo chick hearts, Ca_V_1.2 currents and α1C-subunit expressions at mRNA and protein levels reached the peak at ZT17-20 [23]. However, Ca_V_1.2 currents in mouse cardiomyocytes were larger at ZT3 than that of at ZT15, whereas mRNA and protein expression of α1C-subunit remained constant through the day [11,25]. Ca_V_1.2 channel is highly expressed in VSMCs and is mainly responsible for vascular contraction during excitation-contraction coupling [9]. The present work found that Ca_V_1.2 channels in rat cerebral VSMCs have an obvious circadian rhythm, in which the current densities and α1C-subunit protein expression reached the peak at ZT4 and the trough at ZT16. These results are consistent with the diurnal variation in rat cerebrovascular contractility with the higher at ZT4 and the lower at ZT16, as observed. Furthermore, the present work found that simulated microgravity markedly increased the activities and protein expression at both ZT4 and ZT16, whereas significantly attenuated the diurnal variation in rat cerebral arteries. Interestingly, the Ca_V_1.2 mRNA levels either in CON or in SUS remained relatively constant, which suggests that there might be post-transcriptional regulation mechanisms for Ca_V_1.2 protein expressions. It is noteworthy that the present work showed that there were double bands of Ca_V_1.2 α1c-subunit protein expression in cerebral arteries of CON and SUS rats (Figure 3), whereas single bands of Ca_V_1.2 α1c-subunit protein expression in cultured VSMCs (Figure 4 and Figure 6), which correspond to the predicted size of short and long forms of the Ca_V_1.2 α1c-subunit. We speculate that the double or single band(s) of Ca_V_1.2 α1c-subunit may largely depend on the different samples from different sources and preparations.

Previous studies have shown that both activation of Ras-MAPK (mitogen-activated protein kinase)-Erk (extracellular-signal-regulated kinase) and Ras-PI3K (phosphatidylinositol 3 kinase)- Akt (protein kinase B) are involved in the circadian output pathways in regulating Ca_V_1.2 channels [11,12,13,21,22,24]. Interestingly, calcineurin, NO/cGMP/PKG signaling, AMPK, mTORC1, and REV-ERBα are also reported to be implicated in the circadian regulation of Ca_V_1.2 channels in photoreceptor physiology [12,13,22,24]. In addition to the classic gene regulation, recent studies indicate that miRNAs might play an essential role in the modulation of timekeeping at the post-transcriptional level [23,26,27]. MicroRNAs are a group of small, non-coding, single-stranded RNA and exhibit specific temporal and spatial expression following environmental stimulation. Mature miRNAs could induce one or more downstream target genes’ destabilization and/or translational repression at the post-transcriptional level by biding to 3′-UTR [23,26]. It has been reported that the expression of miR-219 and miR-132 in SCN shows an oscillatory pattern and antagonism of these miRNAs alters the length of circadian period and light-induced resetting of the clock in mice. Inhibition of miR-122 in liver induces post-transcriptional perturbations in the circadian regulation of cholesterol and lipid metabolism. In the present study, we confirmed that miR-103 exhibited an apparent circadian rhythm in rat cerebral arteries, which was in the opposite circadian phase of Ca_V_1.2 protein expression. In addition, simulated microgravity significantly reduced miR-103 expression at both ZT4 and ZT16 in rat cerebral arteries. By vitro targeting reporter assays and gain/loss-function studies, we demonstrated that as a key post-transcriptional regulator, vascular miR-103 directly targeted Ca_V_1.2 α1C-subunit and then negatively modulated Ca_V_1.2 activities and protein expression in a circadian manner.

It has been reported that deletion or mutation of clock gene disrupts cardiovascular circadian rhythms accompanied by dilated cardiomyopathy, arterial stiffness, endothelial dysfunction, impaired cholesterol metabolism, and increased development of atherosclerosis [4,28]. For instance, global deletion of BMAL1 in mice abolished the circadian blood pressure associated with the hypotension. BMAL1 deletion in endothelial cells or VSMCs compromised the diurnal variation of blood pressure. In addition, the mice with deletion of BMAL1 in the heart were more susceptible to arrhythmia and prolonged RR and QRS intervals [29]. On the other hand, cardiovascular diseases may also affect clock gene expression. High salt diet-induced cardiac hypertrophy was associated with attenuated rhythmic expression of core clock genes in rats [30]. In a type 2 diabetic rat model, cardiac clock genes exhibited a phase shift with a 3-h delay [6]. The present study demonstrated that simulated microgravity not only altered the expression of BMAL1, but also attenuated the diurnal variations in both central clock of SCN and peripheral clock of cerebral arteries. Furthermore, BMAL1 was found to induce miR-103 expression in VSMCs, which in turn modulated the protein expression of Ca_V_1.2 channel at the post-transcriptional level. These results validate the signal pathway of BMAL1/miR-103/Ca_V_1.2 was involved in simulating microgravity-induced circadian dysfunction of cerebrovascular contractility. Actually, we have also investigated other clock gene expression at protein and mRNA levels in cerebral arteries isolated from control and simulated microgravity rats, such as Per2 and dbp (data were not shown). However, we could not get enough evidence to demonstrate the relationships of Per2/dbp and Ca_V_1.2 channel in simulated microgravity rats at now.

The occurrence of postflight orthostatic intolerance has been regarded as a major adverse effect and there are still no effective countermeasures until now. Better understanding of the role of circadian regulation in cerebrovascular adaptation will lead to new chronotherapeutic countermeasures during microgravity exposure. In addition, we also want to provide novel theoretical knowledge to understand the relationships between microRNAs and the circadian timing systems. We found that simulated microgravity altered the clock gene BMAL1 in the central clock of SCN and the peripheral clock of cerebral arteries. Therefore, it is suggested that BMAL1-induced miR-103 could be a communicated link between the core clock and peripheral vascular clock output signaling that further governs biological functions which need further research. In summary, our present studies provide compelling evidence that (1) the clock gene BMAL1 could induce the expression of miR-103 and in turn modulate the circadian regulation of Ca_V_1.2 channel in rat cerebral arteries at post-transcriptional level; and (2) simulated microgravity disrupts intrinsic diurnal oscillation in rat cerebrovascular contractility by altering circadian regulation of BMAL1/miR-103/Ca_V_1.2 signal pathway. Our work provides a novel mechanism underlying the circadian dysfunction in cerebrovascular contractility when exposed to microgravity.

## 4. Materials and Methods

### 4.1. Animal Model

Male Sprague-Dawley rats (weight: 200–220 g) were housed individually in controlled environments (21–23 °C, 40–50% humidity) on a 12-h light/12-h darkness cycle (8:00 a.m. to 8:00 p.m.). Zeitgeber time zero (ZT0) was designated as the time when the lights turned on and ZT12 was the time when the lights turned off. The experimental simulated microgravity rat model was successfully established by using modified suspension techniques from our laboratory as described previously [3,9,19], in which rats were maintained in about –30° head-down tilt position with their hindlimbs unloaded to simulate the cardiovascular deconditioning effects of microgravity. All animals received standard rat chow and water ad libitum. At the end of a 28-day simulation period, animals were anesthetized with pentobarbital sodium (50 mg/kg i.p.) and killed by exsanguination via the abdominal aorta. The wet weight of the left soleus was measured to confirm the efficacy of deconditioning. All the samples were collected under normal LD cycles. All procedures complied with the National Institutes of Health Guide for the Care and Use of Laboratory Animals and with IACUC approval at the Fourth Military Medical University. Table of Animal Experimental Ethical Inspection was submitted on 5th September 2015 and approved with the NO. 20150911 by Experimental Animal Ethics and Welfare Committee of Fourth Military Medical University. 

### 4.2. Examination of Vasoconstrictor Responsiveness

To determine the vascular contraction in response to constrictor stimulation, middle cerebral arteries were isolated from CON and SUS rats at zeitgeber time 4 (ZT4) and ZT16. As previously described [19], the segment of middle cerebral artery was transferred to the PSS containing (in mM): 119 NaCl, 4.7 KCl, 1.2 MgSO_4_, 1.2 KH_2_PO_4_, 25 NaHCO_3_, 2.5 CaCl_2_, 5.5 glucose, and 0.026 EDTA, equilibrated with 95% O_2_ and 5% CO_2_ at pH 7.4 adjusted with NaOH. To focus on studying VSMC function, the endothelial layer was mechanically removed by the injection of air bubbles and then cannulated by two pipettes with nylon suture in a vessel chamber. After cannulation, the chamber was transferred to the Pressure Myograph System P110 (DMT, Aarhus, Denmark) and the arterial segment was perfused under a pressure of 25 mmHg for 5–10 min to check the leaking and then remove the blood residue. The arterial segment was allowed to equilibrate at 37 °C and 50 mmHg for 1 h. After equilibration, the arterial viability was evaluated by its reactivity to 20 and 60 mM isotonic KCl. Then the pressure was cycled three times between 25 and 125 mmHg to reduce mechanical hysteresis. Concentration-response relationships were determined by the cumulative superfusion of 5-hydroxytryptamine (5-HT, 10^−10^ to 10^−5^ M) while the arteries were pressurized at 50 mmHg in Ca^2+^-contained PSS. Contractile response to cumulative superfusion of 5-HT was represented as the percentage of luminal diameter relative to the baseline internal diameter according to the formula: Luminal diameter change (%) = (Di,a,s − Di,a,b)/Di,a,b. × 100 %, where Di,a,b was the baseline internal diameter measured in active state at a pressure of 50 mmHg and Di,a,s was the steady-state internal diameter measured to each subsequent change in agonist concentration at the same pressure.

### 4.3. Examination of Myogenic Tone

To determine the vascular contraction in response to mechanical stretch, the myogenic responses were investigated in middle cerebral arteries isolated from SUS and CON rats at ZT4 and ZT16. Intraluminal pressure was increased from 0 to 150 mmHg by increments of 25 mmHg [19]. Each step was maintained for 5–10 min to allow the vessel to reach a steady-state diameter. When vasomotion was present, the steady-state mean diameter was calculated. Finally, a passive pressure-diameter relationship was achieved by incubating the arterioles with Ca^2+^-free PSS containing 2 mM EGTA and sodium nitroprusside (SNP; 0.01 mM) for 30 min and repeated. The myogenic tone was calculated as: Myogenic tone (%) = (Di,p − Di,a)/Di,p × 100%, where Di,p was the passive internal diameter determined in Ca^2+^-free PSS containing 2 mM EGTA and Di,a was the active internal diameter determined in Ca^2+^-contained PSS at a particular intraluminal pressure.

### 4.4. Isolation of Cerebral Arteries and VSMCs

Isolation of cerebral arteries and subsequent enzymatic VSMCs isolation were carried out as previously described [9,31]. Briefly, the brain tissue was carefully removed and placed in 4 °C physiological salt solution (PSS) containing (in mM) 137 NaCl, 5.6 KCl, 1MgCl_2_, 0.42 Na_2_HPO_4_, 0.44 NaH_2_PO_4_, 4.2 NaHCO_3_, and 10HEPES, equilibrated with 95% O_2_ and 5% CO_2_ at pH adjusted to 7.4 with NaOH. The cerebral arteries, including superior, middle, and basilar arteries, with the circle of Willis were dissected out and then harvested. Cerebral arteries were cut into 1–2 mm length and digested for 18 min at 37 °C with solution containing 4 mg/mL papain (Biochrom, Berlin, Germany), 2 mg/mL dithioerythritol (Amresco, St. Louis, MO, USA), 1 mg/mL bovine serum albumin (BSA) (MP Biomedicals, Illkirch, France), and 5 mM taurine in PSS. Following, the segments were transferred to enzyme-free PSS containing 1 mg/mL BSA and 5 mM taurine at room temperature for 10 min and triturated with a flame-polished pipette to disperse VSMCs. Isolated VSMCs were suspended in Ca^2+^-free PSS containing 1 mg/mL BSA and 5 mM taurine and stored at 4 °C for use within 8 h.

### 4.5. Electrophysiological Recordings

Currents were recorded using the whole-cell patch-clamp technique in isolated cerebral VSMCs, with an amplifier (CEZ-2300, Nihon Kohden, Tokyo, Japan) and a version interface (Axon Instruments, Foster City, CA, USA), as described previously [9,31]. Command-voltage protocols and data acquisition were performed with pCLAMP software (version 8.0, Axon Instruments). Patch pipettes (tip resistance 2–6 MΩ when filled with a pipette solution) were fabricated on an electrode puller (Narishige Instruments, Tokyo, Japan) with borosilicate glass capillary tubing. Cell capacitance (Cm) and access resistance were estimated from the capacitive current transient evoked by application of a 20-mV pulse for 40 ms from a holding potential of −60 to −40 mV. To account for differences in cell size, currents were normalized to Cm to obtain the current densities. All measurements were performed at room temperature (25 °C). The cell was held at −40mV and then stepped in 10 mV increments from −40 to +70 mV. Voltage steps were 250 ms in duration, and 2 s intervals were allowed between steps. Currents were filtered at 0.5 kHz and digitized at 4 kHz. Nonspecific membrane leakage and residual capacitive currents were subtracted with the p/4 protocol. Ba^2+^ replaced Ca^2+^ as charge carrier to increase unitary currents and to minimize Ca^2+^-dependent run-down. To obtain the I-V curve of Ca_V_1.2, the current densities were plotted against the corresponding command potentials. Two kinds of external solutions, solutions A and B, were used. Solution A was used while making a gigaohm seal between the recording pipette and cell surface. It contained (in mM) 120 NaCl, 30 Mannitol, 3 K_2_HPO_4_, 1 MgSO_4_, and 30 HEPES, and supplemented with 0.1% bovine serum albumin and 0.5% glucose at pH 7.4 titrated with NaOH. After a seal of 2 GΩ was obtained, the perfusion fluid was changed to solution B during current recording. Solution B contained (in mM) 108 BaCl_2_ and 10 HEPES, pH corrected to 7.6 with BaOH_2_. Cs^+^ was used in the pipette solution to minimize outward K^+^ current. The pipette contained (in mM) 150 CsCl, 5 EGTA, 10 HEPES, 5 Na_2_ATP, and 10 D-glucose at pH 7.2 titrated with CsOH. To identify the properties of Ca_V_1.2, extracellular application of 0.1 μM nifedipine (the specific blocker) and 5 μM Bay K 8644 (the specific agonist) were used in this study.

### 4.6. Cell Culture and Treatment

A7r5 cells (rat thoracic aortic smooth muscle cell line) were purchased from the Type Culture Collection of the Chinese Academy of Sciences (Shanghai, China) and cultured in dulbecco’s modified eagle medium (DMEM) (Hyclone, Utah, USA), supplemented with 10% fetal bovine serum (FBS) (Thermo Scientific, Rockford, IL, USA), 100 U/mL penicillin (Solarbio, Beijing, China), and 100 µg/mL streptomycin (Solarbio, Beijing, China). The cells were maintained in culture at 37 °C under an atmosphere of 5% CO_2_ and subcultured every 48 h.

### 4.7. Protein Extraction and Western Blotting

Rats were anesthetized and sacrificed from ZT0 to ZT20 at 4-h intervals. Cerebral arteries and SCN were separated attentively and then grinded with a glass homogenizer in T-PER Tissue Protein Extraction Reagent (Thermo Scientific) with freshly 1% protease inhibitor cocktail (Thermo Scientific) on ice [9]. A7r5 cells lysates were also prepared in the same lysis buffer. After centrifugation at 12,000× *g* for 10 min at 4 °C, supernatants were used for Western blotting. Total protein concentrations were determined following the instructions with bicinchoninic acid (BCA) Protein Assay Kit (Thermo Scientific). Equivalent amounts of proteins from different groups were loaded to adjacent lanes of NuPAGE 4–12% Bis-Tris gel (Thermo Scientific), and electrophoresed for 50 min at 200 volts. Proteins were transferred to polyvinylidene fluoride (PVDF) membranes (Millipore, Billerica, MA, USA) at 30 volts for 3 h. Membranes were blocked for 4 h using 5% BSA in phosphate buffered saline (PBS) at room temperature, and then incubated with appropriate antibodies on a swing bed at 4 °C overnight. The following antibodies were used: Rabbit anti-BMAL1 polyclonal antibody (1:1000, abcam, Eugene, USA), rabbit anti-Ca_V_1.2 polyclonal antibody (1:200, Alomone Labs, Jerusalem, Israel), and mouse anti-β-actin monoclonal antibody (1:1000, Proteintech, Wuhan, China). The membranes were then incubated for 2 h with horseradish peroxidase (HRP)-conjugated secondary antibodies (1:10,000, zhongshan, Beijing, China), and detected and visualized using the chemiluminescent HRP substrate (Millipore). Software Image J was applied for densitometry measurement.

### 4.8. RNA Extraction and Real-time Quantitative Reverse Transcription PCR (qRT-PCR)

Briefly, cerebral arteries, SCN, and A7r5 cells were mixed and homogenized with RNAiso (Takara, Otsu, Japan) [26]. After incubation in room temperature for 5 min, the mixture was centrifuged at 12,000× *g* for 10 min at 4 °C, chloroform was then added to supernatants for phase separation. Total RNAs, located in the aqueous phase, were precipitated with isopropyl alcohol. After centrifugation, the supernatants were discarded, and RNA pellet was washed with 75% ethanol twice and dried for 10 min at room temperature. Finally, the pellet was dissolved in RNase-free water and stored at −80 °C for further analysis. For the qRT-PCR assay, total RNA, including miRNAs, was reverse transcribed to cDNA by using Mir-X miRNA First-Strand Synthesis Kit (Takara, Otsu, Japan) according to the manufacturer’s protocol. Then, cDNA was amplification with SYBR Premix Ex TaqTM (Takara, Otsu, Japan) using a CFX96 (Bio-rad, Richmond, CA, USA) instrument. Data were analyzed via the relative Ct (2^−ΔΔCt^) method and were expressed as a fold change compared with the respective control. The *Bmal1* cDNA was amplified with a pair of primers (reverse 5’-CCAACCCATACACAGAAGCA-3’ and forward 5’-TTCCCTCGGTCACATCCTAC-3’). The *Cav1.2* cDNA was amplified with a pair of primers (reverse 5’-TGCTGTGTCTGACCCTGAAG-3′ and forward 5’-CGTCTTCCGGAAAGGGAATA-3′). The *β-actin* cDNA was amplified with a pair of primers (reverse 5’-TCAGGTCATCACTATCGGCAAT-3′ and forward 5’-AAAGAAAGGGTGTAAAACGCA-3′).

### 4.9. Computational Prediction of miRNAs Which Targets Ca_V_1.2α1C Subunit

Several potential miRNA related to Ca^2+^ signal were primarily screened by the published literatures and the target prediction software programs of microRNA target and target downregulation, such as miRanda (http://www.microrna.org), miRDB (http://www.mirdb.org), and Targetscan (http://www.targetscan.org). The qRT-PCR was then used to confirm the candidate miRNAs in the cerebral arteries of simulated microgravity rat.

Primers used for real time quantitative RT-PCR were:

miR-328, 5′-ACACTCCAGCTGGGCTGGCCCTCTCTG-3′;
miR-145, 5′-ACACTCCAGCTGGGGTCCAGTTTTCCCAG-3′;
miR-103, 5′-ACACTCCAGCTGGGAGCAGCATTGTACAG-3′;
miR-137, 5′-ACACTCCAGCTGGGTTATTGCTTAAGAA-3′;
miR-1, 5′-ACACTCCAGCTGGGTGGAATGTAAAGAAG-3′;
miR-133a, 5′-ACACTCCAGCTGGGTTTGGTCCCCTTCA-3′;
miR-206, 5′-ACACTCCAGCTGGGTGGAATGTAAGGAA-3′;
miR-26a, 5′-ACACTCCAGCTGGGTTCAAGTAATCC-3′;
U6, 5′-CTCGCTTCGGCAGCACA-3′(forward),
5′-AACGCTTCACGAATTTGCGT-3′(reverse).

### 4.10. Dual-Luciferase Report Assay

PsiCHECK^TM^-2 vector (Promega, Madison, WI, USA) that contains both Firefly and Renilla luciferase genes was used to introduce the wild/mutant 3′-UTR sequences of CACNA1C to stop codon of the Renilla luciferase gene downstream to create a wild-type (WT) or mutant-type (MUT) CACNA1C 3′-UTR plasmid (Sangon Biotech, Shanghai, China). PsiCHECK™-2 vector without inserted gene was used as negative control (NC) plasmid. According to the manufacturer’s protocols, A7r5 cells were seeded in 24-well plates and co-transfected with PsiCHECK^TM^-2 vector (WT/MUT/NC) and miR-103 mimic (100 nmol/L) using the Lipofectamine3000 reagent. The cells were lysed after 48 h of transfection, and both the Firefly and Renilla luciferase activities (Fluc, Rluc) were sequentially measured using the Dual-Luciferase Reporter Assay system (Promega) as recommended. Relative luciferase activity was calculated by normalizing Rluc to Fluc, the value in NC plasmid plus mimic control treated group was set to 1.

### 4.11. Plasmid and Oligonucleotides Transient Transfection

The DNA plasmid GV141-BMAL1 (Genechem, shanghai, China), siRNA targeting against BMAL1 sequence (siBMAL1), and miR-103 mimic/inhibitor were used in the present study. The DNA plasmid and oligonucleotides transfection were performed using Lipofectamine3000 reagent (Invitrogen, Carlsbad, CA, USA) and Opti-MEM Reduced-Serum medium (Invitrogen) as described previously. A7r5 cells were transfected with plasmid or oligonucleotides for 48 h before functional assays were carried out. The sequences were as follows: Bmal1 siRNA, TCTTCAAGATCCTCAATTA; miR-103-3p mimic, 5′-AGCAGCAUUGUACAGGGCUAUCA-3′(sense), 5′-UGAUAGCCCUGUACAAUGCUGCU-3′(antisense); miR-103-3p inhibitor, 5′-UGAUAGCCCUGUACAAUGCUGCU-3′.

### 4.12. Statistical Analysis

Each experiment was performed at least in triplicates. For relative gene expression, the mean value for the control group was defined as 100%. Statistical analysis using unpaired *t*-test (two group comparison), one-way ANOVA or two-way ANOVA, and Tukey’s multiple comparisons test (multiple group comparison) was done with SPSS software. A *p*-value < 0.05 was considered to be statistically significant. Details of the statistical analysis for each experiment are reported in figure legends.

## Figures and Tables

**Table 1:** Body weight and wet weight of the soleus muscle of rats in CON and SUS group.

Group	Body Weight (g, $\bar{x}$ ± SEM)		Wet Weight of the Soleus Muscle ($\bar{x}$ ± SEM)	
	Initial	Final	Absolute (mg)	Ratio (mg/g)
CON	235.4 ± 6.4	394.6 ± 8.3	143.9 ± 3.4	0.37 ± 0.02
SUS	231.8 ± 5.3	386.2 ± 7.1	68.3 ± 2.9 ***	0.18 ± 0.01 ***

CON: 28-day simultaneous control rats; SUS: 28-day tail-suspended rats, *n* = 120, $\bar{x}$ ± SEM, *** *p* < 0.001, as compared with CON.

## Supplementary Materials

Supplementary materials can be found at https://www.mdpi.com/1422-0067/20/16/3947/s1.

## Abbreviations

SUS	tail-suspended hindlimb-unweighting
SCN	suprachiasmatic nuclei
VSMCs	vascular smooth muscle cells
CCGs	clock controlled genes
HRV	heart rate variability
ZT	zeitgeber time
