# Supplementary material for: BMAL1 Disrupted Intrinsic Diurnal Oscillation in Rat Cerebrovascular Contractility of Simulated Microgravity Rats by Altering Circadian Regulation of miR-103/CaV1.2 Signal Pathway
Source: Int J Mol Sci. 2019 Aug 14;20(16):3947. doi: 10.3390/ijms20163947 (PMC6720455; doi:10.3390/ijms20163947)
Supplement: Supplementary file 1 [file ijms-20-03947-s001.pdf]

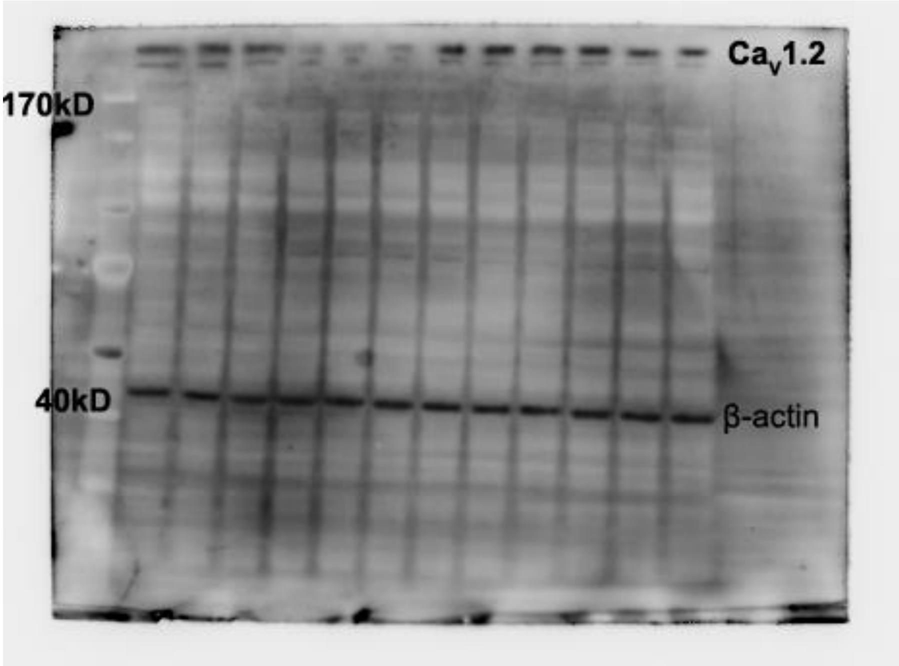

Full-length Western blots for Figure 3A

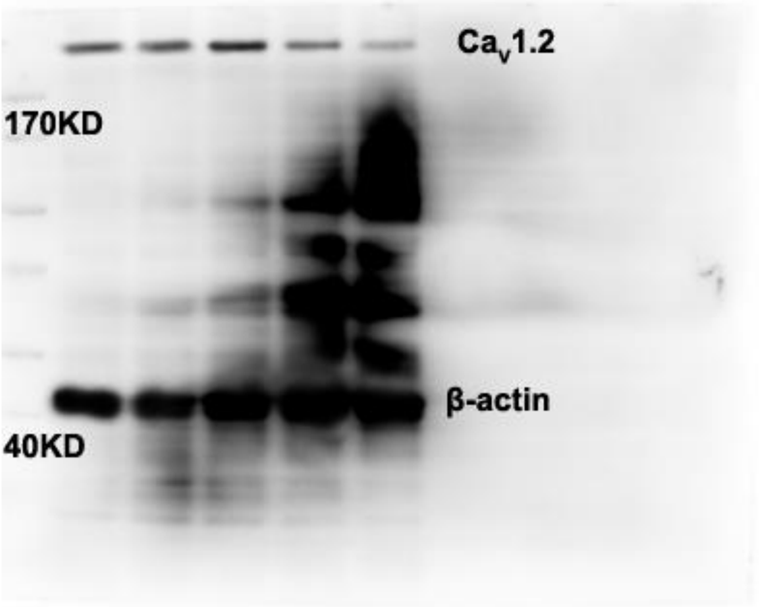

Full-length Western blots for Figure 4F

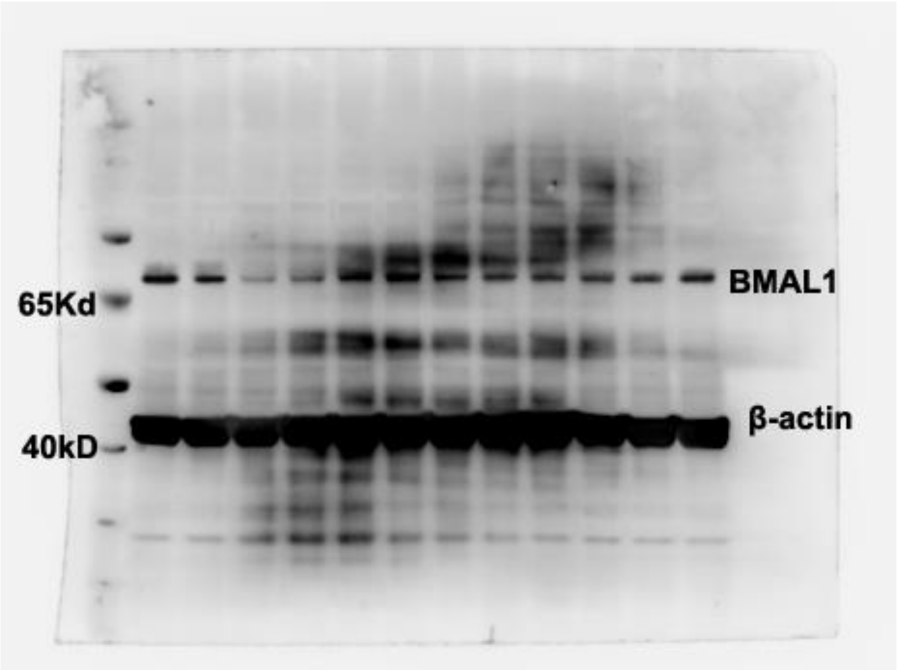

Full-length Western blots for Figure 5A

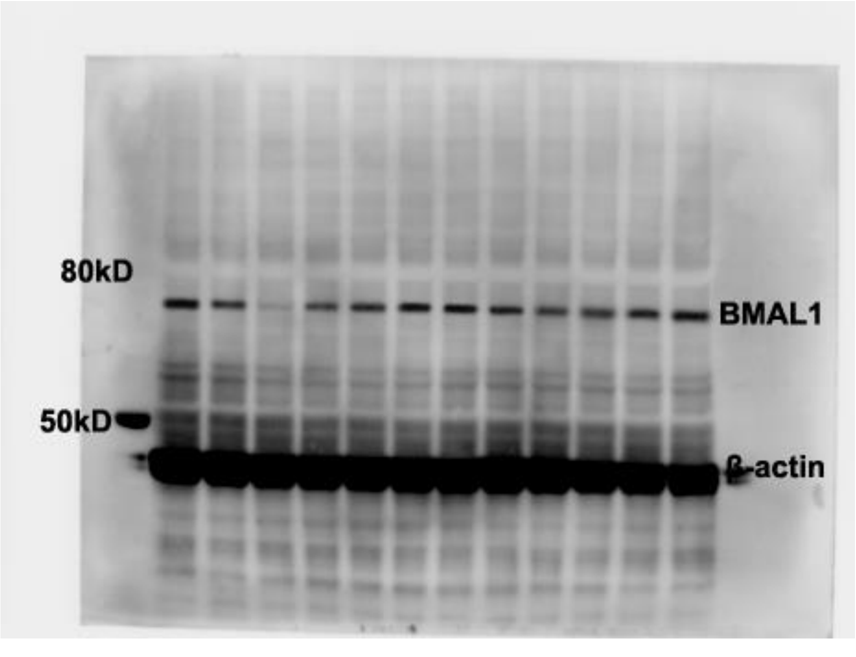

Full-length Western blots for Figure 5F

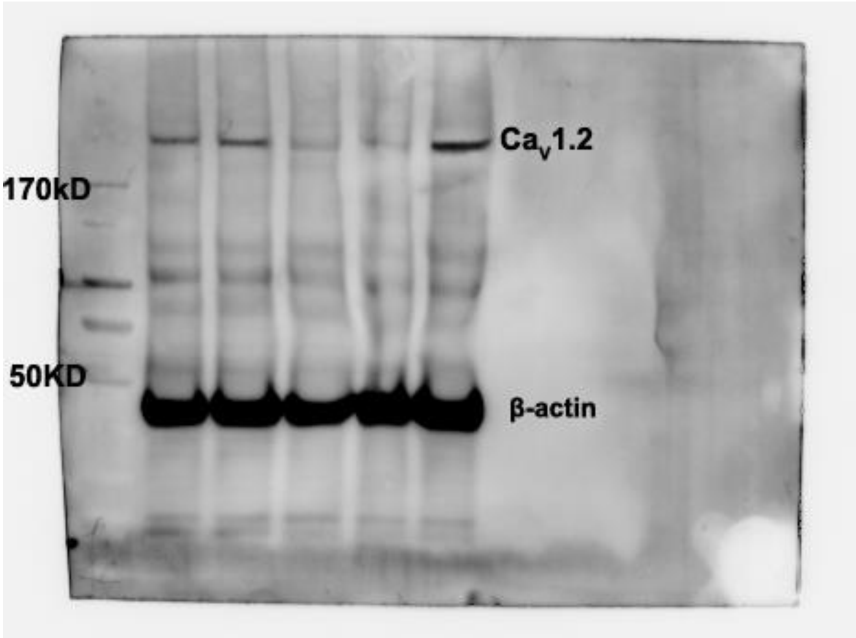

Full-length Western blots for Figure 6B

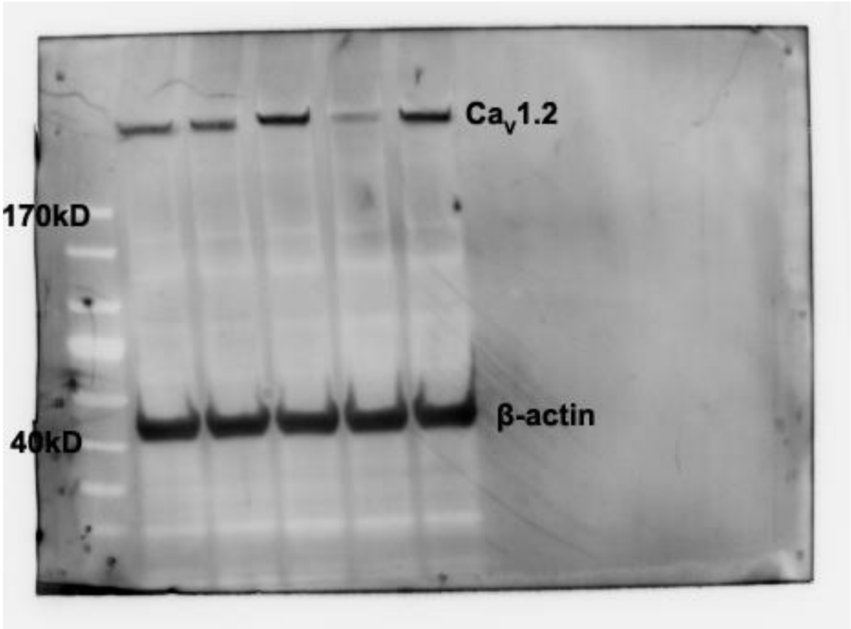

Full-length Western blots for Figure 6F
